# Noninvasive assessment of clinical and pathological characteristics of patients with IgA nephropathy by diffusion kurtosis imaging

**DOI:** 10.1186/s13244-022-01158-y

**Published:** 2022-01-29

**Authors:** Ping Liang, Shichao Li, Guanjie Yuan, Kangwen He, Anqin Li, Daoyu Hu, Zhen Li, Chuou Xu

**Affiliations:** Department of Radiology, Tongji Hospital, Tongji Medical College, Huazhong University of Science and Technology, 1095 Jiefang Avenue, Wuhan, 430030 Hubei China

**Keywords:** Diffusional kurtosis imaging, Immunoglobulin A nephropathy, Mean kurtosis, Mean diffusivity, Apparent diffusion coefficient

## Abstract

**Objectives:**

To explore the diagnostic performance of diffusion kurtosis imaging (DKI) in evaluating the clinical and pathological characteristics of patients with immunoglobulin A nephropathy (IgAN) compared with conventional DWI.

**Materials and methods:**

A total of 28 IgAN patients and 14 healthy volunteers prospectively underwent MRI examinations including coronal T2WI, axial T1WI, T2WI, and DWI sequences from September 2020 to August 2021. We measured mean kurtosis (MK), mean diffusivity (MD), and apparent diffusion coefficient (ADC) by using MR Body Diffusion Toolbox v1.4.0 (Siemens Healthcare). Patients were divided into three groups according to their estimated glomerular filtration rate (eGFR) (Group1, healthy volunteers without kidney disease or other diseases that affect renal function; Group2, IgAN patients with eGFR > 60 mL/min/1.73 m^2^; Group3, IgAN patients with eGFR < 60 mL/min/1.73 m^2^). One-way analysis of variance, Pearson or Spearman correlation, and receiver operating characteristic curves were applied in our statistical analysis.

**Results:**

MK_Cortex_ and ADC_Cortex_ showed significant differences between the Group1 and Group2. MK_Cortex_, MD_Cortex_, ADC_Cortex_, MK_Medulla_, and ADC_Medulla_ showed significant differences between Group2 and Group3. MK_Cortex_ had the highest correlation with CKD stages (*r* = 0.749, *p* < 0.001), and tubulointerstitial lesion score (*r* = 0.656, *p* < 0.001). MD_Cortex_ had the highest correlation with glomerular lesion score (*r* = − 0.475, *p* = 0.011). MK_Cortex_ had the highest AUC (AUC = 0.923) for differentiating Group1 from Group2, and MD_Cortex_ had the highest AUC (AUC = 0.924) for differentiating Group2 from Group3, followed by MK_Medulla_ (AUC = 0.923).

**Conclusions:**

DKI is a feasible and reliable technique that can assess the clinical and pathological characteristics of IgAN patients and can provide more valuable information than conventional DWI, especially MK_Cortex_.

## Key points

- DKI may be a feasible technique to evaluate the clinical and pathological characteristics of patients with IgAN.
- MK of the renal cortex derived from DKI may provide more valuable information than ADC.
- MK of the renal cortex may be an excellent parameter for the evaluation of early kidney changes in patients with IgAN.

## Introduction

Immunoglobulin A nephropathy (IgAN) is the most common glomerular disease in the world, which is characterized by the presence of IgA dominant or codominant immune deposits in the glomeruli [1]. The incidence of IgA nephropathy is 2.5 cases per 100,000 adults per year, and its prevalence is always underestimated because not all patients with suspected renal insufficiency will undergo a kidney biopsy [2]. IgAN is a chronic and progressive disease with various clinical manifestations (from asymptomatic to gross hematuria) and nearly 14–39% patients will develop end stage renal disease (ESRD) within 20 years after diagnosis [3]. Histopathological findings, including glomerular sclerosis, renal tubular atrophy and interstitial fibrosis, are the main independent risk factors for predicting the progression of IgAN and are essential for clinical treatment and prognostic evaluation [4]. However, kidney biopsy is an invasive and traumatic procedure, which may cause complications such as bleeding and infection. Moreover, it is difficult to repeat biopsy for longitudinal monitoring due to sampling errors [5]. Therefore, it is necessary to find a noninvasive method with high reproducibility and easy implementation in clinical practice to evaluate the pathological and clinical characteristics of IgAN.

A Working Group of the International IgA Nephropathy Network and the Renal Pathology Society reported that initial estimated glomerular filtration rate (eGFR) based on creatinine and urinary protein excretion at the time of renal biopsy are the clinical parameters which are the risk factors of IgAN and are associated with renal prognosis [6]. However, the sensitivity and specificity of the eGFR for early assessment of renal function are limited, because it represents the overall function of both kidneys, and the kidneys have a strong compensatory ability in the early stage [7]. Previous study also showed that most chronic kidney disease patients with proteinuria less than 1 g/24 h can be classified as low-risk and can only be treated through outpatient follow-up, while patients with high-risk (proteinuria greater than 1 g/24 h) need to be registered and treated through inpatient management [8]. However, the measurements of proteinuria need to collect 24 h urine, which is inconvenient, especially for outpatients.

With the development of magnetic resonance imaging (MRI) equipment hardware and software, diffusion weighted imaging (DWI) is increasingly used in the evaluation of diseases in abdominal organs [9–11]. Conventional DWI calculates the apparent diffusion coefficient (ADC) to quantify the random motion of water molecules in biological tissues, but this monoexponential model assumes that water molecules follow a Gaussian statistical distribution [12]. However, the cell membranes and intracellular organelles of the tissues in vivo will cause the non-Gaussian diffusion of water molecule, resulting in ADC not being able to accurately reflect the true diffusion of water molecules in the complex microenvironment. Based on this background, diffusion kurtosis imaging (DKI), which include multiple *b*-values and ultra-high *b*-values (> 1000 s/mm^2^), is introduced as a non-Gaussian diffusion model to evaluate the true structural information of the tissue [13]. Previous study demonstrated that DKI can provide quantitative parameters such as mean kurtosis (MK), mean diffusivity (MD) to quantify the deviation of water diffusion from Gaussian distribution [14]. Previous studies on breast cancer, rectal cancer, cervical cancer have shown the potential of DKI to provide more accurate water molecular diffusion information in vivo, and it can more accurately evaluate the stages of tumors [15–17]. Recently, a study demonstrated that DKI can evaluate renal fibrosis in patients with IgAN [18]. However, this study only applied renal cortex DKI parameters and did not have control group.

The purpose of this study was to evaluate the clinical and pathological characteristics of patients with IgAN by using the renal cortex and medulla DKI parameters compared with conventional DWI.

## Materials and methods

### Patients

**Fig. 1:**
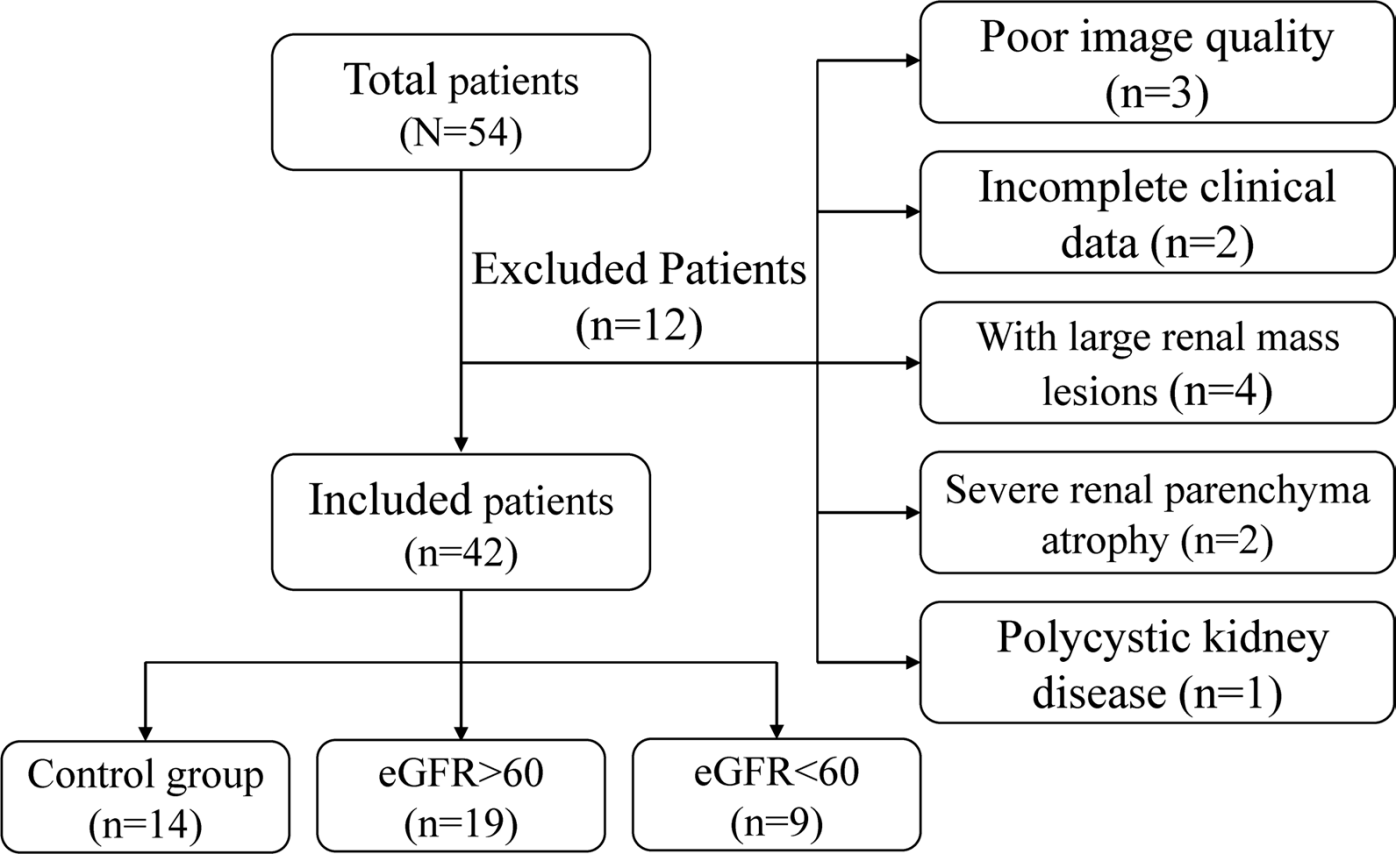
Flowchart of the study population

The ethics committee of our hospital approved this prospective study and written informed consent was obtained from all participants. All patients from the Department of Nephrology who were suspected of having IgAN underwent MRI scans before renal biopsy. We enrolled 54 patients with chronic kidney disease (CKD), forty of whom were pathologically confirmed as IgAN from December 2020 to July 2021. Twelve patients with IgAN were excluded according to the following criteria: 1, poor image quality (*n* = 3); 2, incomplete clinical data (*n* = 2); 3, large renal mass lesions (*n* = 4); 4, severe renal parenchyma atrophy (*n* = 2); 5, polycystic kidney disease (*n* = 1). Finally, twenty-eight patients with pathologically confirmed IgAN were included in this study (15 male, 13 female; mean age, 38.61 ± 10.12 years; range, 22–58 years). We subsequently included 14 healthy volunteers of similar age and gender without kidney disease or other diseases that affect renal function (6 male, 8 female; mean age, 44.86 ± 13.65 years; range, 27–66 years). Patients were divided into three groups according to their eGFR (Group1, healthy volunteers; Group2, IgAN patients with eGFR > 60 mL/min/1.73 m^2^; Group3, IgAN patients with eGFR < 60 mL/min/1.73 m^2^). Inclusion criteria are presented in Fig. 1.

### Laboratory parameters

All patients underwent intravenous blood sampling to assess renal function, including serum creatinine (Scr), uric acid, blood urea nitrogen (BUN). The normal values of Scr, uric acid, and BUN are 59–104 umol/L, 202–416 umol/L, 3.1–8.0 mmol/L, respectively. The eGFR was calculated based on the modification of diet in renal disease (MDRD) formula [19]:

$${\text{eGFR}}\,{\text{(mL/min/}}1.73\,{\text{m}}^{2} {)} = 186 \times ({\text{Scr}})^{ - 1.154} \times ({\text{Age}})^{ - 0.203} \times (0.742\;{\text{if}}\;{\text{Female}}) \times (1.210\;{\text{if}}\;{\text{African}}\;{\text{American}})$$

All blood biochemical tests were performed within 1 week of renal biopsy and MRI.

### Pathology parameters

All patients underwent ultrasound-guided renal biopsy within 1–2 days after completing the renal MR examinations and the biopsy site was the lower pole of the right kidney in our study. All kidney specimens were obtained by percutaneous ultrasound-guided kidney biopsy and sent for immunofluorescence, optical microscopy, and electron microscopy pathological evaluation. This analysis process is similar to the previous study [20] and was completed by a nephrologist with 15 years of clinical experience in our hospital. We calculated the glomerular, tubulointerstitial, and vascular lesion scores by using Katafuchi semi-quantitative standards [21]. The glomerular lesion scores ranged from 0 to 12 points including glomerular cell proliferation (0–4 points), segmental lesions (0–4 points), and glomerular sclerosis (0–4 points). The tubulointerstitial lesion scores ranged from 0 to 9 points including interstitial fibrosis (0–3 points), tubular atrophy (0–3 points), and interstitial inflammatory cell infiltration (0–3 points). The vascular lesion scores ranged from 0 to 6 points including vascular thickening (0–3 points), and hyaline degeneration (0–3 points). The details of the pathology scores are shown in Table 1.

**Table 1:** A semi-quantitative standard for calculating the scores of glomerular, tubular interstitial and vascular lesions

Scores	Glomerular lesion score			Tubulointerstitial lesion score			Vascular lesion score	
	Glomerular cell proliferation (%)	Segmental lesions (%)	Glomerular sclerosis (%)	Interstitial fibrosis (%)	Tubular atrophy (%)	Interstitial inflammatory cell infiltration (%)	Vascular thickening (%)	Hyaline degeneration (%)
1	≤ 25	≤ 10	≤ 10	≤ 25	≤ 25	≤ 25	≤ 10	≤ 25
2	25–50	10–25	10–25	25–50	25–50	25–50	10–25	25–50
3	50–75	25–50	25–50	≥ 50	≥ 50	≥ 50	≥ 25	≥ 50
4	≥ 75	≥ 50	≥ 50	NA	NA	NA	NA	NA

NA, not applicable

### MRI acquisition

All MRI examinations were completed within 1–2 days before the kidney biopsy and all patients including volunteers were informed fast for 8 h and water for 4 h before the MR examinations. All patients will be informed of safety precautions and relieve their nervousness before the examination and perform breathing training according to our instructions. The MRI examinations were performed on a 3T scanner (MAGNETOM Skyra, Siemens Healthcare, Erlangen, Germany) with an eighteen-channel phased-array coil. Conventional coronal T2WI, axial T1WI, T2WI, and a prototype DKI-DWI sequences were performed. DKI-DWI applied a single-shot echoplanar imaging (EPI) sequence during free-breathing and combined with reduced field-of-view (ZOOMit) with tilted excitation plane in axial orientation [22]. The parameters were as follows: FOV = 288 × 125 mm, slice thickness = 5.0 mm, Matrix = 120 × 120, TR = 7700 ms, TE = 72 ms. We applied fat saturation technology to reduce chemical shift artifacts and applied a 4-directional diffusion-weighting gradient that included 5 *b*-values (0, 500, 1000, 1500, 2000). The acquisition time ranged from 4 to 5 min, varying based on the number of slices.

### Image analysis

**Fig. 2:**
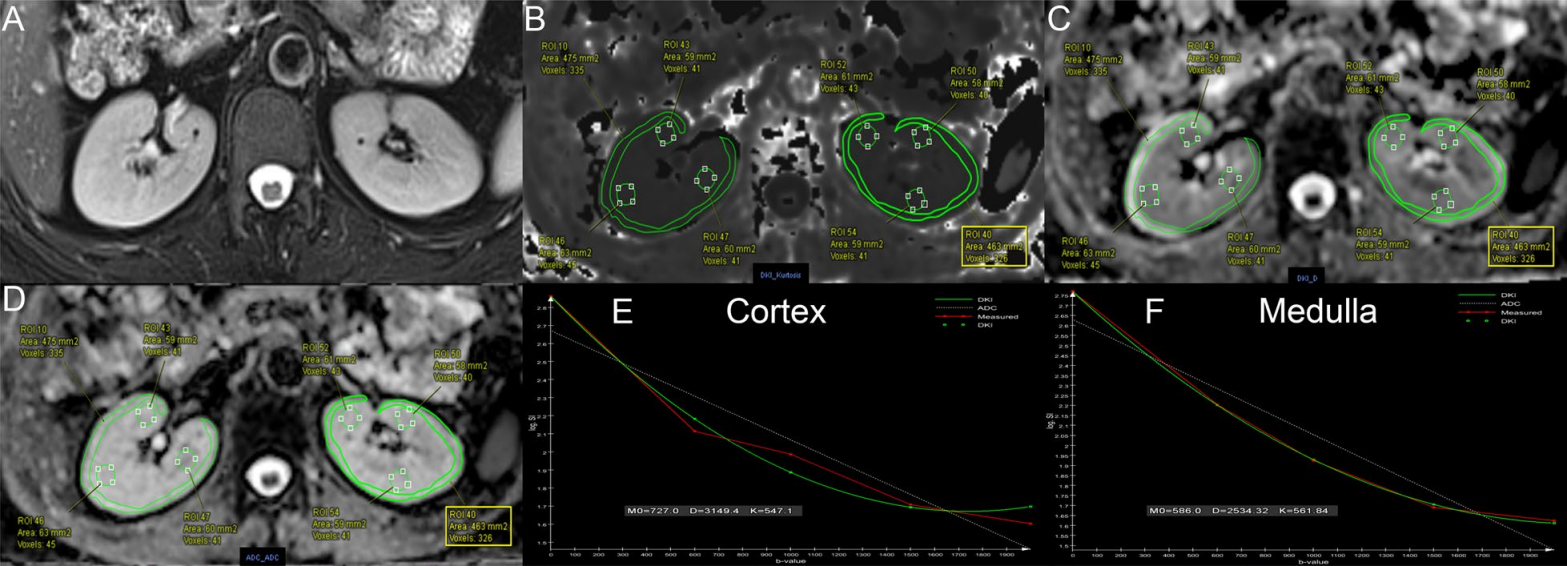
Respective MRI images of healthy volunteers with eGFR = 109.5 mL/min/1.73 m^2^. **A**–**F** axial T2-weighted image, MK, MD, ADC, cortex DKI fit, medulla DKI fit, respectively

**Fig. 3:**
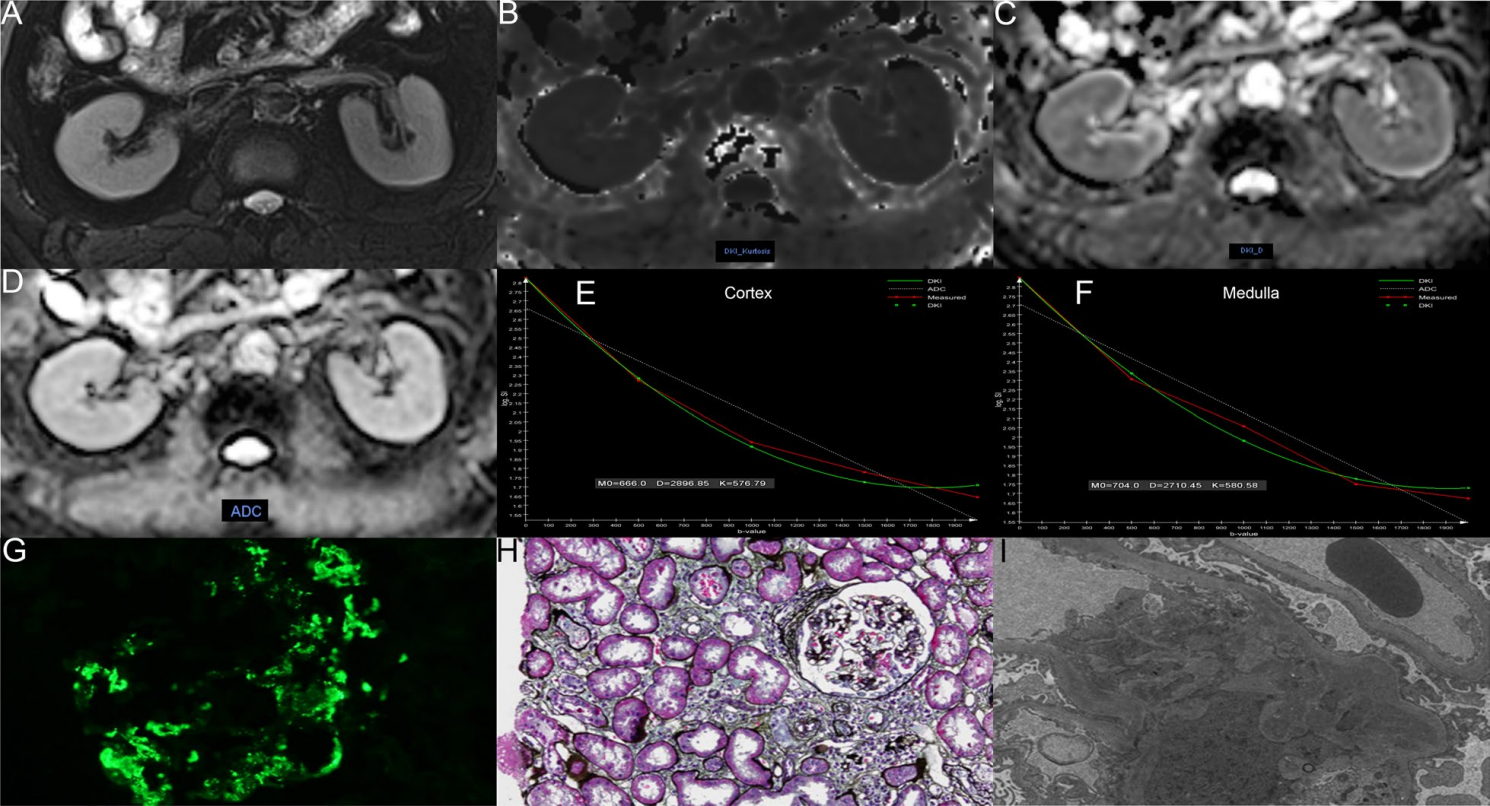
Respective MRI and pathological images of the IgAN patients with eGFR = 77.4 mL/min/1.7 3m^2^. **A**–**I** axial T2-weighted image, MK, MD, ADC, cortex DKI fit, medulla DKI fit, immunofluorescence, light microscopy, and electron microscopy, respectively. The pathological result of this patient is M0E0S1T1C1

**Fig. 4:**
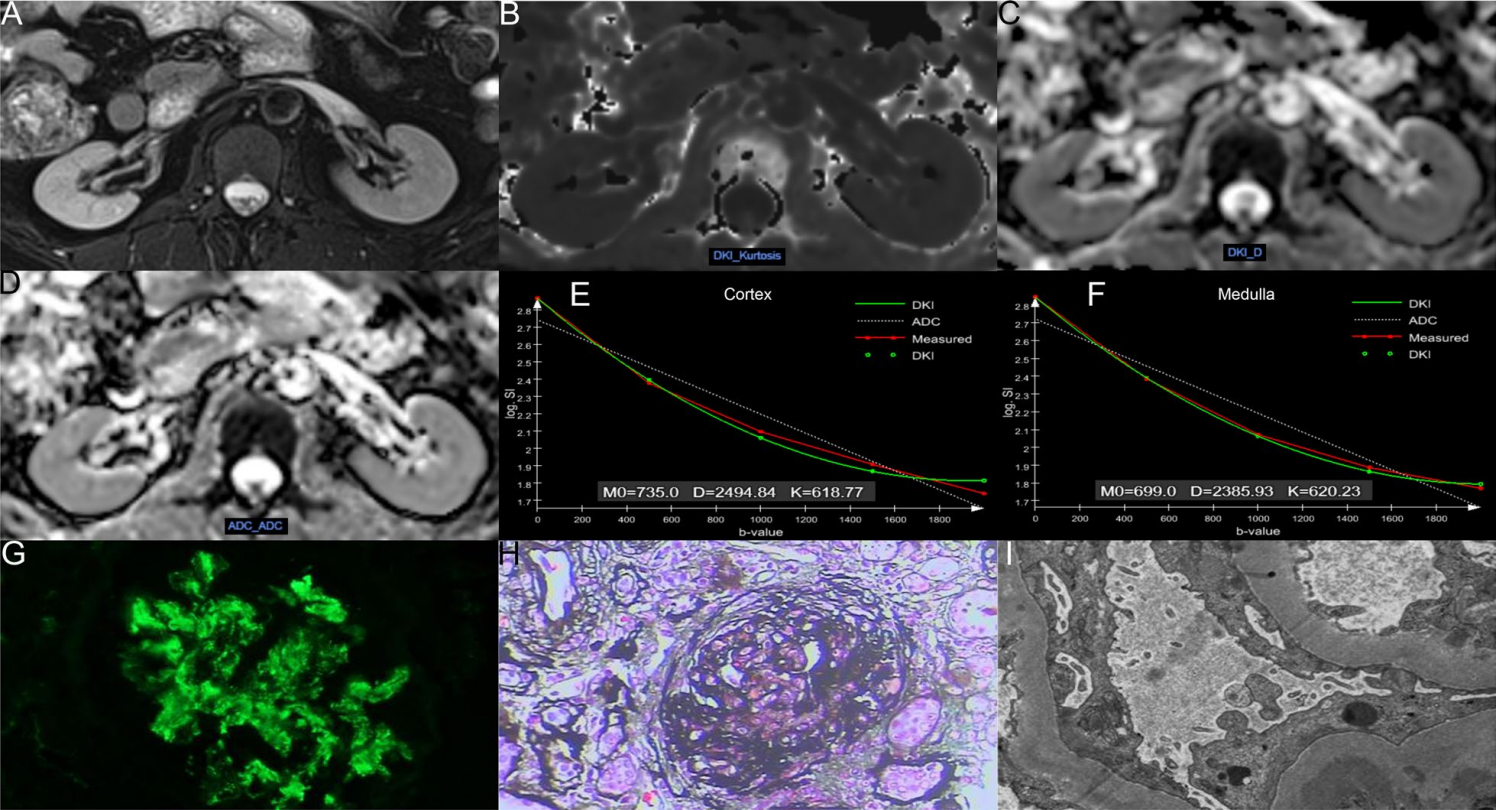
Respective MRI and pathological images of the IgAN patients with eGFR = 18.7 mL/min/1.73 m^2^. **A**–**I** axial T2-weighted image, MK, MD, ADC, cortex DKI fit, medulla DKI fit, immunofluorescence, light microscopy, and electron microscopy, respectively. The pathological result of this patient is M0E1S1T2C0

We transferred the original images from the workstation to our hard disk and used the post-processing software offline provided by MR Body Diffusion Toolbox v1.4.0 (Siemens Healthcare, Erlangen, Germany) to obtain DKI-DWI parameters (MK, MD) and ADC values. Two radiologists with 8 and 18 years of experience in abdominal imaging, without knowing the clinical information of the patients, delineated the bilateral renal cortex and medullary regions of interest (ROIs) at the largest level through the renal hilum. Cortical ROIs (4.51 ± 0.59 cm^2^) were drawn along the outline of the kidneys avoiding large vessels, fat, and cysts. Three medullary ROIs (0.61 ± 0.02 cm^2^) were delineated on each kidney by using the T2WI anatomical images as a reference. The placement of cortical and medullary ROIs is shown in Fig. 2, which shows the MRI image of healthy volunteers with eGFR = 109.5 mL/min/1.73 m^2^. Figures 3 and 4 show the MRI and pathological images of the IgAN patients with eGFR = 77.4 mL/min/1.73 m^2^ and eGFR = 18.7 mL/min/1.73 m^2^, respectively. The values of cortical MK, MD, and ADC in Fig. 2 are 0.547, 3.149 × 10^–3^ mm^2^/s, and 1.948 × 10^–3^ mm^2^/s, respectively. The values of medullary MK, MD, and ADC in Fig. 2 are 0.562, 2.534 × 10^–3^ mm^2^/s, and 1.755 × 10^–3^ mm^2^/s, respectively. The values of cortical MK, MD, and ADC in Fig. 3 are 0.576, 2.897 × 10^–3^ mm^2^/s, and 1.821 × 10^–3^ mm^2^/s, respectively. The values of medullary MK, MD, and ADC in Fig. 3 are 0.581, 2.710 × 10^–3^ mm^2^/s, and 1.711 × 10^–3^ mm^2^/s, respectively. The values of cortical MK, MD, and ADC in Fig. 4 are 0.618, 2.494 × 10^–3^ mm^2^/s, and 1.624 × 10^–3^ mm^2^/s, respectively. The values of medullary MK, MD, and ADC in Fig. 4 are 0.620, 2.385 × 10^–3^ mm^2^/s, and 1.589 × 10^–3^ mm^2^/s, respectively. ADC values were calculated by using a monoexponential model with b-values of 0 and 1000 s/mm^2^ according to the following equation [23]:

1 $$Sb = S0 \times e^{{( - b \times ADC_{mon)} }}$$

where *Sb* is the signal at a given *b* value (*b* = 1000 s/mm^2^ in our study), *S*0 is the signal when *b* = 0 s/mm^2^.

MK and MD were calculated with all of *b* values (0–2000s/mm^2^) by using the following equation [24]:

2 $$Sb = S0 \times e^{{\left( { - b \times D + 1/6 \times b2 \times D2 \times K} \right)}}$$

where *Sb* is the signal at a particular *b* value, *S*0 is the signal when *b* = 0 s/mm^2^. *D* represents the ADC analog adjusted for non-Gaussian diffusion behavior and the unit is mm^2^/s. *K* represents the excess kurtosis relative to a monoexponential fit and has no unit. It is 0 in tissues with complete Gaussian diffusion and increases with greater deviation from the Gaussian pattern.

### Statistical analysis

All statistical analyses were performed on SPSS (version 22, Chicago, IL) and the values of *p* < 0.05 were considered statistically significant. We used Shapiro–Wilk test to evaluate the normality of data distribution (*p* ≥ 0.05 demonstrates normal distribution). Normally distributed variables were expressed as means ± standard deviations. One-way analysis of variance (ANOVA) was used to compare the differences of clinical and DKI parameters among the three groups. The least-significant difference (LSD) test was used to compare the differences between any two groups. The intraclass correlation coefficients (ICCs) were employed to evaluate the interobserver agreements (0.81–1.00, excellent agreement; 0.61–0.80, moderate agreement; 0.21–0.40, fair agreement; 0.00–0.20, poor agreement). Pearson product-moment correlation was used to evaluate the relationships between the diffuse parameters and clinical parameters and Spearman's rank correlation was used to evaluate the relationships between the diffuse parameters and pathological scores (The absolute value of the correlation coefficient below 0.3 represented no linear correlation, 0.3–0.5 was low correlation, 0.5–0.8 was moderate correlation, and above 0.8 was highly correlated). Receiver operating characteristic (ROC) curves were used to evaluate the diagnostic performance of DKI parameters for differentiating Group2 from Group1, or Group3.

## Results

### Clinical characteristics

Fourteen healthy volunteers and 28 IgAN patients were included in the statistical analysis. There were no significant differences in sex (*p* = 0.798) among the three groups. However, the age between the Group1 and Group2 showed significant difference (*p* = 0.038), and the age of Group2 is significantly smaller than that of Group1. The values of eGFR, Scr, uric acid, and BUN showed significant differences among the three groups (all *p* < 0.001). Furthermore, the values of Scr, uric acid, and BUN increase with the decrease in eGFR. The details of these clinical parameters are shown in Table 2.

**Table 2:** Baseline clinical data and diffusional kurtosis imaging (DKI) parameters

Characteristics	Control group	eGFR ≥ 60 mL/min	eGFR < 60 mL/min	*P*^α^	*P*^*β*^	*P*^*γ*^
*Clinical data*						
No. of patients	14	19	9			
Males/females	6/8	10/9	5/4	0.579	0.552	0.885
Age, year	44.86 ± 13.65	36.37 ± 9.49	43.33 ± 10.28	0.038	0.752	0.132
eGFR, mL/min/1.73 m^2^	106.47 ± 9.79	89.20 ± 22.62	35.53 ± 13.82	0.008	< 0.001	< 0.001
SCr, μmol/L	58.00 ± 16.68	82.79 ± 20.21	190.11 ± 49.45	0.016	< 0.001	< 0.001
Uric acid, mg/dL	247.71 ± 106.26	326.66 ± 90.67	430.88 ± 91.31	0.025	< 0.001	0.011
BUN, mg/dL	4.11 ± 1.61	5.74 ± 1.38	9.18 ± 2.98	0.019	< 0.001	< 0.001
*DKI metrics*						
MK_Cortex_	0.536 ± 0.011	0.571 ± 0.028	0.629 ± 0.053	0.002	< 0.001	< 0.001
MD_Cortex_	3.031 ± 0.320	2.963 ± 0.235	2.553 ± 0.207	0.462	< 0.001	< 0.001
ADC_Cortex_	1.937 ± 0.142	1.836 ± 0.105	1.702 ± 0.084	0.017	< 0.001	0.006
MK_Medulla_	0.564 ± 0.030	0.581 ± 0.027	0.633 ± 0.025	0.100	< 0.001	< 0.001
MD_Medulla_	2.592 ± 0.363	2.691 ± 0.319	2.459 ± 0.198	0.378	0.327	0.076
ADC_Medulla_	1.744 ± 0.101	1.737 ± 0.086	1.632 ± 0.113	0.843	0.010	0.010

eGFR, estimated glomerular filtration rate; SCr, serum creatinine; BUN, blood urea nitrogen; MK, mean kurtosis; MD, mean diffusivity; ADC, apparent diffusion coefficient

^α^Represents the comparison of parameters between control group and eGFR ≥ 60 mL/min

^β^Represents the comparison of parameters between control group and eGFR < 60 mL/min

^γ^Represents the comparison of parameters between eGFR ≥ 60 mL/min and eGFR < 60 mL/min

### Interobserver agreement

The interobserver agreements of the renal cortex and medulla MK, MD, and ADC between the two radiologists are excellent (all ICC > 0.80). The results showed that the reproducibility of the diffuse parameters was excellent, and it was suitable for repeated measurements for long-term longitudinal follow-up of patients with IgAN. Therefore, we randomly selected the measurement results of one radiologist for the statistical analysis. The ICC values and 95% confidence intervals (95% CI) of the renal cortex and medulla MK, MD, and ADC are present in Table 3.

**Table 3:** The interobserver agreements between two radiologists of the diffusional kurtosis imaging (DKI) metrics

Parameters	ICC	95% CI
MK_Cortex_	0.978	0.959–0.988
MD_Cortex_	0.969	0.942–0.983
ADC_Cortex_	0.967	0.939–0.982
MK_Medulla_	0.913	0.836–0.953
MD_Medulla_	0.924	0.858–0.959
ADC_Medulla_	0.899	0.813–0.946

MK, mean kurtosis; MD, mean diffusivity; ADC, apparent diffusion coefficient; ICC, intraclass correlation coefficient; CI, confidence intervals

### Comparisons of the diffuse parameters between different groups

**Fig. 5:**
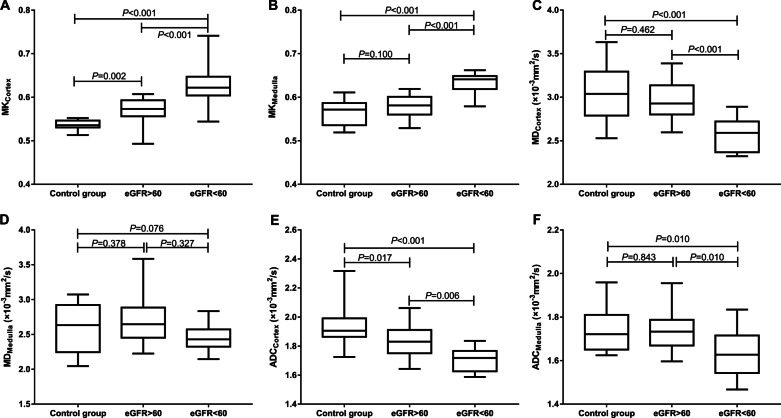
Comparison of cortical and medullary diffusion parameters between the three groups

Only MK_Cortex_ and ADC_Cortex_ showed significant differences between the Group1 and Group2 (*p* = 0.002, *p* = 0.017, respectively). MK_Cortex_, MD_Cortex_, ADC_Cortex_, MK_Medulla_, and ADC_Medulla_ showed significant differences between Group1 and IgAN patients with eGFR < 60 mL/min/1.73 m^2^ (*p* < 0.001, *p* < 0.001, *p* < 0.001, *p* < 0.001, and *p* = 0.010, respectively). There were also significant differences in MK_Cortex_, MD_Cortex_, ADC_Cortex_, MK_Medulla_, and ADC_Medulla_ between Group3 and Group2 (*p* < 0.001, *p* < 0.001, *p* = 0.006, *p* < 0.001, and *p* = 0.010, respectively). MD_Medulla_ showed no significant differences between Group1 and Group3, or Group2 and Group3. These results are present in Table 2 and Fig. 5.

### Correlations between diffuse parameters and clinicopathological parameters

**Fig. 6:**
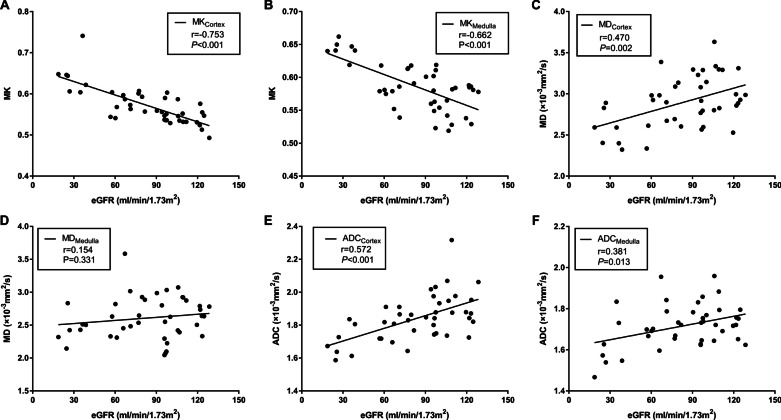
Correlations between cortical and medullary diffusion parameters and eGFR

**Fig. 7:**
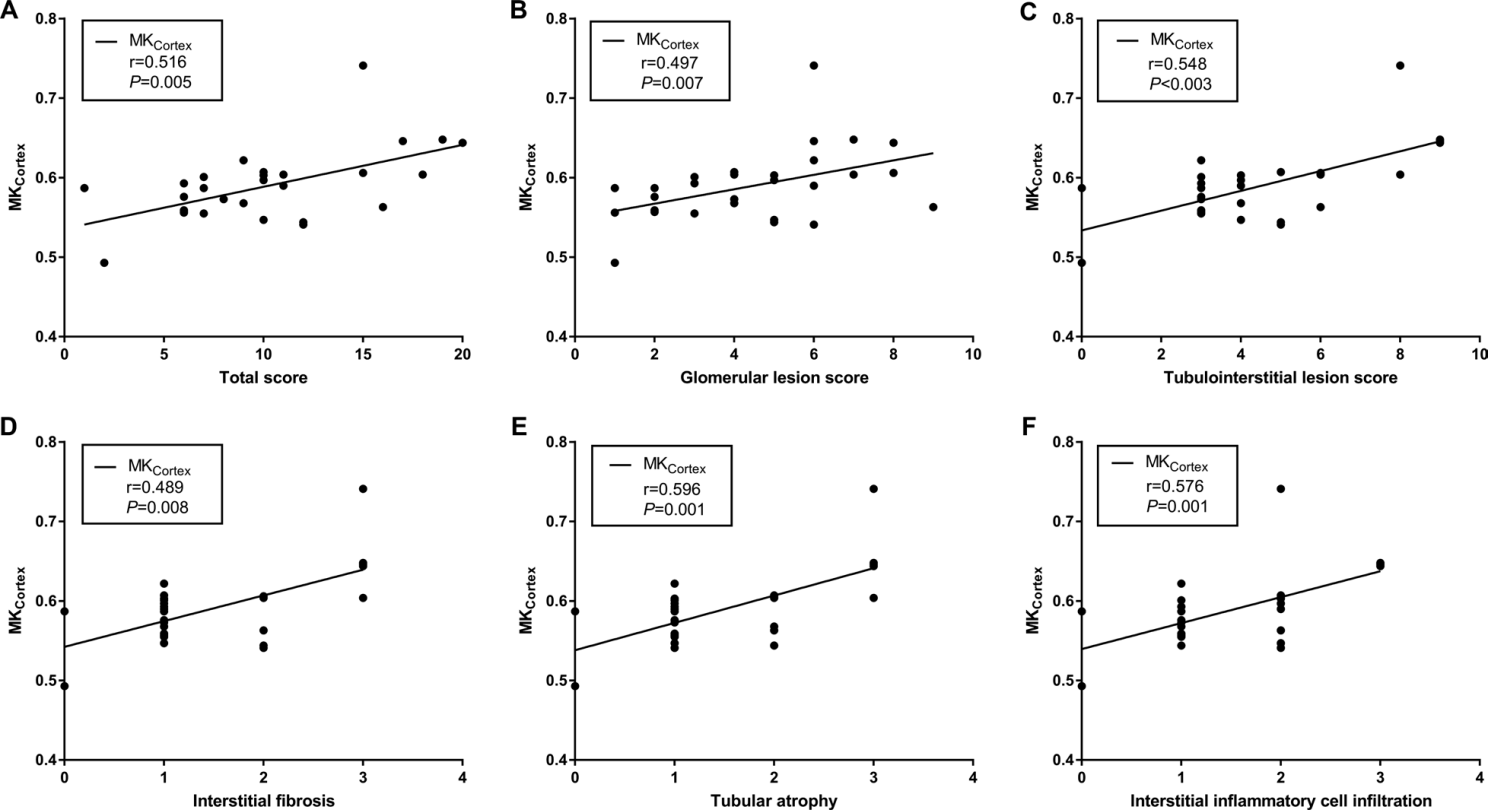
Correlations between MK_Cortex_ and pathology score

MK_Cortex_ and MK_medulla_ showed moderate correlations with CKD stages, eGFR, Scr, uric acid, and BUN. MD_Cortex_ showed low correlations with CKD stages, eGFR, Scr, uric acid, and BUN. MD_Medulla_ showed no significant correlations with clinical parameters. ADC_cortex_ showed low to moderate correlations with CKD stages, eGFR, Scr, uric acid, and BUN. ADC_Medulla_ showed low correlations with CKD stages, eGFR, Scr. There were no significant correlations between ADC_Medulla_ and uric acid, or BUN. Furthermore, MK_Cortex_ and Scr showed the highest correlation coefficient (*r* = 0.781, *p* < 0.001). The correlation coefficients and correlation scatter plot between the diffusion parameters and clinical parameters are shown in Table 4 and Fig. 6, respectively. MK_Cortex_, MK_Medulla_, MD_Cortex_, and ADC_Cortex_ also showed low to moderate correlations with pathology score, such as total score, glomerular lesion score, tubulointerstitial lesion score, interstitial fibrosis, tubular atrophy, and interstitial inflammatory cell infiltration. MD_Medulla_ only showed low negative correlations with total score, glomerular lesion score, tubulointerstitial lesion score, and tubular atrophy. ADC_Medulla_ showed no correlation with glomerular lesion score. The highest correlation coefficients between the diffusion parameters and pathology scores were MK_Cortex_ and tubular atrophy (*r* = 0.596, *p* = 0.001). The correlation coefficients and correlation scatter plot between the diffusion parameters and clinical parameters are shown in Table 4 and Fig. 7, respectively.

**Table 4:** Correlations between the diffusional kurtosis imaging (DKI) metrics and clinicopathological data

	MK				MD				ADC			
	Cortex		Medulla		Cortex		Medulla		Cortex		Medulla	
	*r*	*P*	*r*	*P*	*r*	*P*	*r*	*P*	*r*	*P*	*r*	*P*
CKD stages	0.749	< 0.001	0.686	< 0.001	− 0.499	0.001	− 0.092	0.563	− 0.635	< 0.001	− 0.419	0.006
eGFR	− 0.753	< 0.001	− 0.662	< 0.001	0.470	0.002	0.154	0.331	0.572	< 0.001	0.381	0.013
SCr	0.781	< 0.001	0.752	< 0.001	− 0.485	0.001	− 0.135	0.394	− 0.606	< 0.001	− 0.440	0.004
Uric- acid	0.485	0.001	0.496	0.001	− 0.397	0.009	− 0.180	0.253	− 0.425	0.005	− 0.224	0.153
BUN	0.730	< 0.001	0.587	< 0.001	− 0.322	0.038	− 0.122	0.441	− 0.400	0.009	− 0.296	0.057
Total score	0.516	0.005	0.437	0.023	− 0.506	0.006	− 0.410	0.030	− 0.532	0.004	− 0.251	0.197
Glomerular lesion score	0.497	0.007	0.430	0.025	− 0.509	0.006	− 0.398	0.036	− 0.442	0.018	− 0.273	0.159
Tubulointerstitial lesion score	0.548	0.003	0.499	0.008	− 0. 471	0.011	− 0.398	0.036	− 0.541	0.003	− 0.261	0.179
Vascular lesion score	0.135	0.493	0.177	0.378	− 0.130	0.508	− 0.094	0.634	− 0.219	0.262	− 0.094	0.635
Interstitial fibrosis	0.489	0.008	0.545	0.003	− 0.526	0.004	− 0.269	0.166	− 0.513	0.005	− 0.173	0.379
Tubular atrophy	0.596	0.001	0.536	0.004	− 0.487	0.009	− 0.370	0.053	− 0.438	0.020	− 0.189	0.334
Interstitial inflammatory cell infiltration	0.576	0.001	0.482	0.011	− 0.316	0.102	− 0.287	0.139	− 0.546	0.003	− 0.245	0.210

MK, mean kurtosis; MD, mean diffusivity; ADC, apparent diffusion coefficient

### Diagnostic performance of the diffusion parameters

**Fig. 8:**
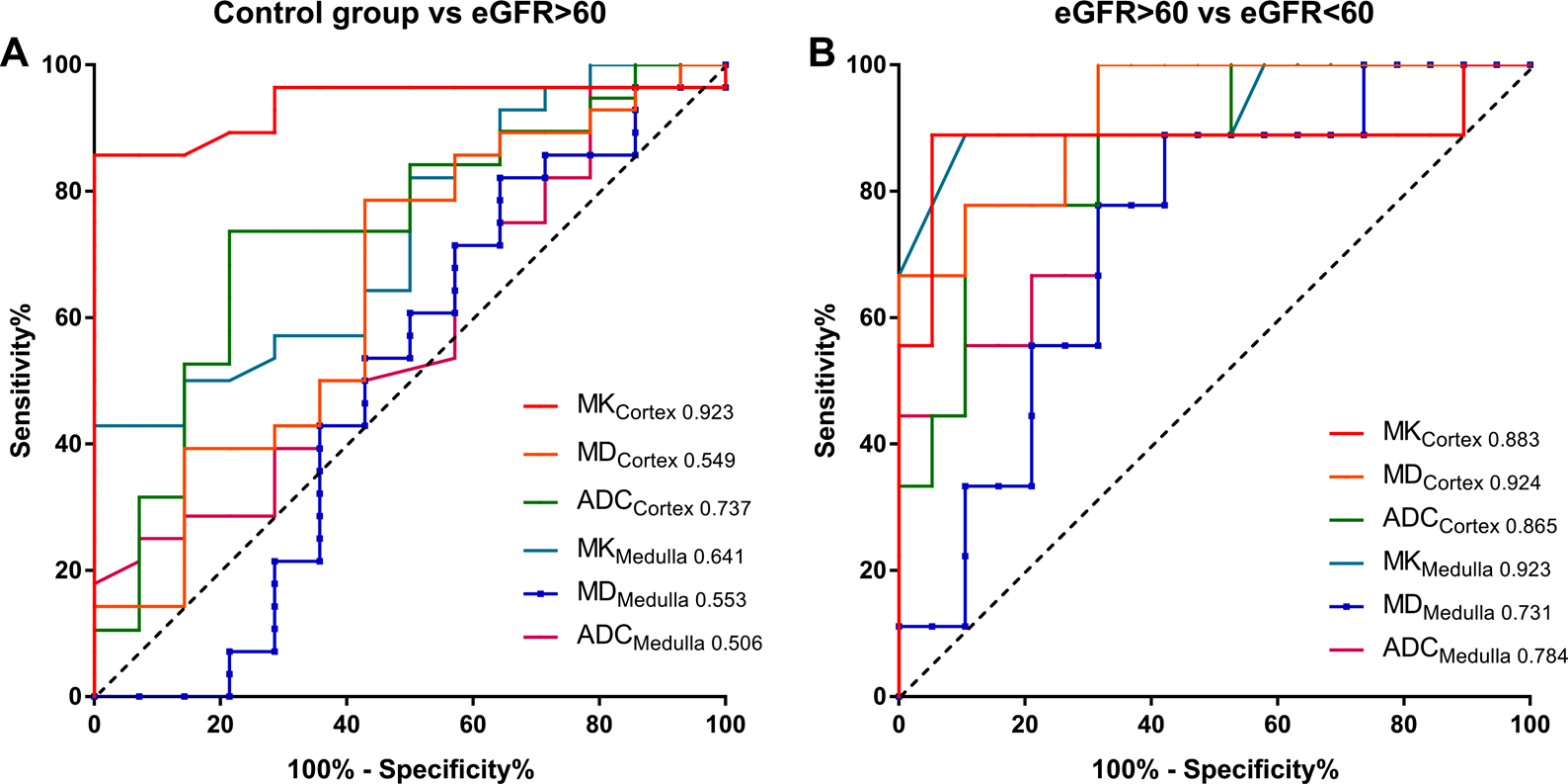
The ROC curve analysis of cortical and medullary diffusion parameters to distinguish between IgAN patients with eGFR > 60 mL/min/1.73 m^2^ and control group, or IgAN patients with eGFR < 60 mL/min/1.73 m^2^

We applied ROC curves analysis to evaluate the diagnostic performance of the different diffusion parameters for differentiating Group2 from Group1 or Group3. The biggest area under the curve (AUC) for differentiating Group2 from Group1 was MK_Cortex_ (AUC, 0.923, 95% CI 0.775–0.987) with sensitivity of 84.21%, specificity of 100%, and a cut-off value of 0.552. Both MD_Cortex_ and MK_Medulla_ showed excellent ability for differentiating Group2 from Group3 (AUC, 0.924, 95% CI, 0.759–0.990; AUC, 0.923, 95% CI 0.753–0.990, respectively) with sensitivity of 100%, 88.89; specificity of 68.42%, 88.89; and a cut-off value of 2.891 mm^2^/s, and 0.552, respectively. The results of the ROC analysis of the diffusion parameters are shown in Table 5 and Fig. 8.

**Table 5:** The results of receiver operating characteristic curves analysis by using renal cortex and medulla MK, MD, and ADC

Parameters	Control group versus eGFR > 60					eGFR > 60 versus eGFR < 60				
	AUC (95% CI)	Cut-off	Sensitivity (%)	Specificity (%)	You-index	AUC (95% CI)	Cut-off	Sensitivity (%)	Specificity (%)	You-index
MK_Cortex_	0.923 (0.775–0.987)	0.552	84.21	100	0.842	0.883 (0.705–0.973)	0.603	88.89	94.74	0.836
MD_Cortex_	0.549 (0.367–0.722)	2.987	68.42	57.14	0.257	0.924 (0.759–0.990)	2.891	100	68.42	0.684
ADC_Cortex_	0.737 (0.555–0.874)	1.866	73.68	78.57	0.523	0.865 (0.684–0.964)	1.727	77.78	89.47	0.673
MK_Medulla_	0.641 (0.453–0.802)	0.563	77.78	50.00	0.279	0.923 (0.753–0.990)	0.614	88.89	88.89	0.779
MD_Medulla_	0.553 (0.370–0.725)	2.295	94.74	28.57	0.233	0.731 (0.531–0.880)	2.632	88.89	57.89	0.468
ADC_Medulla_	0.506 (0.327–0.683)	1.842	5.26	78.57	0.162	0.784 (0.588–0.916)	1.731	88.89	57.89	0.468

MK, mean kurtosis; MD, mean diffusivity; ADC, apparent diffusion coefficient; ROC, receiver operating characteristic; AUC, area under the receiver operating characteristic curve; CI, confidence interval

## Discussion

Our study demonstrated that the non-Gaussian DKI model can effectively evaluate the clinical and pathological characteristics of IgAN, especially MK_Cortex_. MK_Cortex_ was significantly correlated with CKD stages, interstitial fibrosis and renal tubular atrophy, and can well distinguish Group2 from Group1, or Group3.

The interobserver agreements of the renal cortex and medulla MK, MD, and ADC were excellent between the two radiologists. The results showed that the reproducibility and reliability of DKI were convincing, and it can safely, noninvasively and non-radiately carry out long-term longitudinal follow-up of IgAN patients.

MK_Cortex_ showed significant differences among the three groups, even in Group2 and Group1 in our study. However, there was no significant difference in MK_Medulla_ between Group1 and Group2. Furthermore, MK_Cortex_ showed highest significant difference between the Group1 and Group2 among all the diffusion parameters in our study. The results indicated that MK_Cortex_ may be a reliable indicator for early evaluation of IgAN patients and this was consistent with the previous research [25], which showed that renal cortex MK values have the highest sensitivity for detecting changes of renal function in patients with CKD. The MK_Cortex_ increased with the decrease in eGFR and the values of MK_Medulla_ also had a tendency to increase as eGFR decreased. Furthermore, the values of MK_Cortex_ were smaller than MK_Medulla_ in the three groups. This may be due to the more complex microstructures of renal medulla, such as renal tubules and collecting tubules, which make the diffusion of water molecules deviate from Gaussian distribution. These results were consistent with the previous study which demonstrated the values of the renal cortex and medulla MK tended to increase with the decrease in eGFR [26]. MK represents the deviation from Gaussian distribution and previous study showed that MK of tissues represented the interaction of water molecules with cell membranes and intracellular compounds [27, 28]. Sun et al. demonstrated that the more complex the microstructure environment, the greater the values of MK [17]. Liu et al. showed that MK increased with the progression of renal function and the accumulation of collagen fibers [18]. MK_Cortex_ and MK_Medulla_ were not only significantly related to clinical indicators such as eGFR, CKD stages, and Scr, but also significantly related to the glomerular lesion score, and tubulointerstitial lesion score in our study. The results were similar to previous studies [29]. This may be due to deterioration of renal function, leading to glomerular sclerosis, glomerular cell proliferation, and interstitial fibrosis, which will increase the complexity of the renal parenchymal structure and cause the diffusion of water molecules to deviate from the Gaussian distribution. MK represented the complexity of the microenvironment, which will cause the values of MK to increase with decreased renal function and increased pathological scores.

MD is another parameter of DKI, which represents the corrected diffusion coefficient of ADC under non-Gaussian conditions [30]. There was no significant difference in MD_Cortex_ between Group1 and Group2. MD_Cortex_ showed significant differences between Group3 and Group1, or Group2. The results showed that MD_Cortex_ was not sensitive to early changes in IgAN. Zhou et al. showed that MD can also discriminate early diabetic nephropathy from controls [26]. The reason for the differences may be due to the different pathological types and different research objects. However, MD_Cortex_ also had a tendency to decrease from Group1 to Group2 in our study. There were no significant differences in MD_Medulla_ among the three groups, which was similar with the previous study [18]. The values of MD_Cortex_ were larger than MD_Medulla_ in the three groups. This may be due to the more complex microstructures of renal medulla than cortex, leading to restricted diffusion of water molecules. Moreover, MD_Cortex_ had more diagnostic value than MD_Medulla_ in distinguishing Group2 from Group3 in our study. This may be due to the gradual deterioration of renal function, resulting in the gradual replacement of normal glomerular capillaries and tubular structures with extracellular matrix and fibrotic tissue [31]. The effect of this change on the medulla may be smaller than that of the cortex, which was richer in blood flow, and the diffusion of water molecules in the cortex was more restricted than in the medulla [25].

ADC_Cortex_ showed significant differences among the three groups; however, there was no significant difference in ADC_Medulla_ between Group1 and Group2 in our study. Both ADC_Cortex_ and ADC_Medulla_ showed significant correlations with CKD stages, glomerular lesion score, and tubulointerstitial lesion score. Previous study demonstrated that ADC was significantly correlated with split renal function [32] and Zhao et al. showed that renal ADC values were strongly related to histological measurement of fibrosis [33]. These studies showed similar results and ADC may be another excellent indicator for noninvasive evaluation of the clinical and pathological characteristics of IgAN patients.

We used ROC analysis to evaluate the diagnostic performance of the diffuse parameters. The best parameter for differentiating Group1 from Group2 was MK_Cortex_ with the AUC 0.923, followed by ADC_Cortex_ (AUC, 0.737). Furthermore, MD_Cortex_ (AUC, 0.924) was the best parameter for differentiating Group2 from Group3, followed by MK_Medulla_ (AUC, 0.923). The results were consistent with previous study which demonstrated medullary MK was the best parameter for discrimination of the early diabetic nephropathy from the controls without diabetes, followed by cortex MK [26]. These results indicated that glomerular sclerosis, glomerular cell proliferation, and interstitial fibrosis gradually replaced the renal normal structure with the progress of IgAN, causing the diffuse of water molecules to deviate from the Gaussian distribution, and the movement of water molecules was more restricted. MK and MD parameters based on DKI may be good indicators for assessing the progress of IgAN, and ADC also has a certain value in evaluating the renal function in patients with IgAN.

There were several limitations in this study. First, this was a single center research, and the number of IgAN patients were relatively small. Second, the measurement position of ROIs was inconsistent with the biopsy site. We delineated the ROIs through the largest level of the kidney hilum, and the kidney biopsy site was located at the lower pole of the right kidney. We supposed that the deviation was relatively small, because IgAN was a chronic diffuse disease that may affect the entire kidney. Third, Group1 were not age-matched to Group2, which may have a certain impact on our research. Fourth, the standardizations of the acquisition and analysis protocols related to diffusion parameters used in this study are still challenging because they are different in different institutions and different machines.

In conclusion, this study demonstrated that DKI was a feasible and reliable technique that can assess the clinical and pathological characteristics of patients with IgAN, especially cortical MK. Furthermore, DKI can provide more valuable information than conventional DWI and can provide useful information for clinical patient management, treatment, and prognosis.

## Data Availability

The data and materials of this article are available.

## Abbreviations

ADC: Apparent diffusion coefficient
AUC: Area under the receiver operating characteristic curve
BUN: Blood urea nitrogen
DKI: Diffusion kurtosis imaging
DWI: Diffusion weighted imaging
eGFR: Estimated glomerular filtration rate
ESRD: End stage renal disease
ICC: Intraclass correlation coefficient
IgAN: Immunoglobulin A nephropathy
MD: Mean diffusivity
MK: Mean kurtosis
ROC: Receiver operating characteristic
ROI: Region of interest
Scr: Serum creatinine
